# Deciphering the genomic structure, function and evolution of carotenogenesis related phytoene synthases in grasses

**DOI:** 10.1186/1471-2164-13-221

**Published:** 2012-06-06

**Authors:** Bianca Dibari, Florent Murat, Audrey Chosson, Véronique Gautier, Charles Poncet, Philippe Lecomte, Ingrid Mercier, Hélène Bergès, Caroline Pont, Antonio Blanco, Jérôme Salse

**Affiliations:** 1 INRA - UMR 1095 ‘Génétique Diversité Ecophysiologie des Céréales’ (GDEC), 5 Chemin de Beaulieu, 63100, Clermont-Ferrand, France; 2 INRA - Centre National de Ressources Génomiques Végétales (CNRGV), Chemin de Borde Rouge BP 52627, 31326, Castanet Tolosan cedex, France; 3 DIBCA - Department of Agro-Forestry and Enviromental Biology and Chemistry, Sezione di Genetica e Miglioramento Genetico, Via Amendola 165/A, 70126, Bari, Italy

**Keywords:** Carotenoids, Phytoene synthase, Wheat, Intron loss, Abiotic stress, Evolution

## Abstract

**Background:**

Carotenoids are isoprenoid pigments, essential for photosynthesis and photoprotection in plants. The enzyme phytoene synthase (PSY) plays an essential role in mediating condensation of two geranylgeranyl diphosphate molecules, the first committed step in carotenogenesis. PSY are nuclear enzymes encoded by a small gene family consisting of three paralogous genes (*PSY*1-3) that have been widely characterized in rice, maize and sorghum.

**Results:**

In wheat, for which yellow pigment content is extremely important for flour colour, only *PSY*1 has been extensively studied because of its association with QTLs reported for yellow pigment whereas *PSY*2 has been partially characterized. Here, we report the isolation of bread wheat *PSY*3 genes from a *Renan* BAC library using *Brachypodium* as a model genome for the *Triticeae* to develop Conserved Orthologous Set markers prior to gene cloning and sequencing. Wheat *PSY*3 homoeologous genes were sequenced and annotated, unravelling their novel structure associated with intron-loss events and consequent exonic fusions. A wheat *PSY*3 promoter region was also investigated for the presence of *cis*-acting elements involved in the response to abscisic acid (ABA), since carotenoids also play an important role as precursors of signalling molecules devoted to plant development and biotic/abiotic stress responses. Expression of wheat PSYs in leaves and roots was investigated during ABA treatment to confirm the up-regulation of *PSY*3 during abiotic stress.

**Conclusions:**

We investigated the structural and functional determinisms of PSY genes in wheat. More generally, among eudicots and monocots, the PSY gene family was found to be associated with differences in gene copy numbers, allowing us to propose an evolutionary model for the entire PSY gene family in Grasses.

## Background

Carotenoids represent a diverse group of pigments found in bacteria, fungi and plants [1]. In plants, carotenoids have several important functions, essential for plant development as: (*i*) they serve as accessory pigments to harvest light for photosynthesis and constitute the basic structural units of photosynthetic apparatus and (*ii*) they also act as photoprotectors for plants to adapt to high light stress [2]. In addition, oxidative cleavage of carotenoids produces apocarotenoids considered as (*i*) signals in plant development, (*ii*) antifungal agents, (*iii*) contributors to flower and fruit flavours and aromas [3]. The phyto-hormone abscisic acid, the most well known apocarotenoid, plays an important role in plant response to stress conditions [3]. In addition, carotenoids have long been considered as essential nutrients as well as important health beneficial compounds. In animals including humans, unable to synthesize carotenoids *de novo*, dietary carotenoids are essential precursors of vitamin A and retinoid compounds needed in development [4,5].

**Figure 1:**
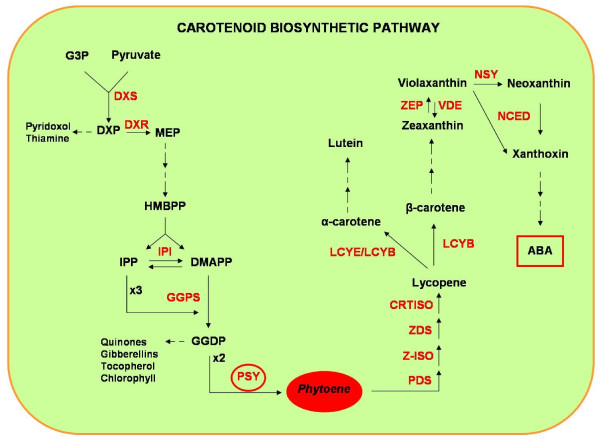
**Carotenoid biosynthetic pathway (adapted from Lu*et al.*2008).** The isopentenyl diphosphate (IPP) is synthesized via the 2-C-methyl-D-erythritol 4-phosphate (MEP) pathway. IPP and DMAPP form the central intermediate geranylgeranyl diphosphate (GGDP) for carotenoid biosynthetic pathway. Phytoene synthase (PSY) acts at the condensation of two molecules of GGDP to form the phytoene, the first step of the carotenoid pathway. Names of compounds are highlighted in black and of enzymes in red. Abbreviations: G3P, glyceraldheyde-3-phosphate; DXP, 1-deoxy-D-xylulose 5-phosphate; HMBPP, 1-hydroxy-2-methyl-2-butenyl 4-diphosphate; ABA, abscisic acid; DXR, 1-deoxy-D-xylulose 5-phosphate reductoisomerase; DXS, 1-deoxy-D-xylulose 5-phosphate; IPI, isopentenyl diphosphate isomerase; GGPS, geranylgeranyl diphosphate synthase; PDS, phytoene desaturase; Z-ISO, ζ-carotene isomerase; ZDS, ζ-carotene desaturase; CRTISO, carotene isomerase; LCYB, β-cyclase; LCYE, ϵ-cyclase; ZEP, zeaxanthin epoxidase; VDE, violaxanthin de-epoxidase; NSY, neoxanthin synthase; NCED, 9-*cis*-epoxycarotenoid dioxygenase.

Plant carotenogenesis occurs in the membrane of chloroplasts, chromoplasts and amyloplasts, plastids having different internal membrane architectures. The enzymes involved in the carotenoid biosynthetic pathway are nuclear encoded and targeted to the plastids. Carotenoid biosynthesis begins with the formation of the 40-carbon backbone, phytoene, a step mediated by phytoene synthase *(*PSY) (*cf* Figure 1) [6,7]. PSY, which is known to control carotenoid flux in seeds and to catalyze the first committed step in carotenogenesis, is thought to be a rate-limiting enzyme. In “Golden Rice” [8], for example, PSY activity was crucial to obtain increased carotenoid levels [9], whereas bacterial carotene desaturase CrtI had no effect [10]. PSY is coded by a small gene family, which had been already shown to exist throughout the Grasses [5] and which consists of three paralogous genes that have been identified and characterized in rice, maize and sorghum. The three rice PSY genes share the same structure, with six exons and five introns, and they exhibit a tissue-specific expression. Rice endosperm is carotenoid free and transcripts for all three genes are absent. In photosynthetic tissues, all three PSY mRNAs are present, but with different levels of expression, Os*PSY*1 and Os*PSY*2 playing predominant roles with similar expression patterns and regulation in response to light. Os*PSY*3 plays a specialized role as it is not regulated by light but is strongly inducible in roots by high salt concentrations and/or drought. Os*PSY*1 and Os*PSY*2 contain *cis*-acting elements involved in light regulation which are absent from the promoter region of Os*PSY*3, explaining why *PSY*3 does not respond to light [11]. Li *et al.*[12] described the PSY gene family and their tissue-specific transcript patterns in maize. They showed that *PSY*1 is the only gene family member for which transcript level increased during maize endosperm development and was correlated with accumulation of endosperm carotenoids. Transcript changes of PSYs were monitored in maize leaves during de-etiolation. In dark-grown plants, *PSY*1 represented the major transcript whereas *PSY*2 was the only paralog for which transcript levels increased in response to illumination, suggesting then that *PSY*2 plays an important role in controlling leaf carotenogenesis during greening. The abundance of *PSY*3 in roots suggests that it plays a unique role in roots carotenogenesis when induced by drought, salt and exogenous ABA treatment. The increase of *PSY*3 transcripts observed was associated with an induced level of carotenoid intermediates, and elevation of other downstream carotenogenic genes, followed by ABA accumulation [12].

PSY genes are of primary importance in wheat due to their impact on flour colour, so that numerous genetics studies have been conducted on yellow pigment (YP) content in wheat grain [13]. A high YP concentration is a desirable trait in durum wheat and is the target of breeding programs worldwide [14]. However, low YP is a breeding target in hexaploid wheat, for which bright white flour is preferred. The inheritance of YP is complex, controlled by additive gene effects and highly heritable [15]. Several QTLs for YP have been identified in both durum and bread wheat on chromosomes 1A [16], 1B [17,18], 3A [19], 3B [16,20], 4A and 5A [21], 2A, 4B and 6B [13], 5B [16], and 6A [17]. However, the majority of studies agreed that chromosome group 7 has the greatest impact in controlling YP content in bread wheat [13,16-19,22-24]. Elouafi *et al.*[22] identified two minor QTLs on chromosome 7A and a third major QTL on chromosome 7BL. This last QTL has been corroborated in two other durum wheat populations [13,24] and is ortholog to the one identified in hexaploid wheat on the same chromosome [25]. A QTL for YP in durum wheat was identified by Patil *et al.*[16] on chromosome 7A that accounted for up to 55% of the trait variation. Mares and Campbell [20] identified QTLs on chromosome 7A in two hexaploid wheat populations that had a large impact on flour yellowness, and correspond to loci also associated with xanthophyll concentration. Cloning of genes controlling flour pigmentation would be of great help in breeding for genotypes with appropriate levels of yellow pigments in durum and bread wheat. Using a PCR-based approach Cenci *et al.*[26] identified and mapped clones from a durum wheat BAC library thought to contain genes coding for important enzymes involved in carotenogenesis. In their study, putative phytoene synthase, phytoene desaturase and ζ-carotene desaturase clones were assigned to the chromosome groups 5, 4 and 2, respectively. To date, four PSY genes have been identified in durum wheat, forming two paralogous series on chromosome groups 5 (*Psy*2) and 7 (*Psy*1) [13]. One of these, *Psy*1-B1, co-segregates with a QTL for YP on 7BL [13,24]. *Psy*1-A1 was localized on the distal end of the chromosome 7AL in one hexaploid wheat mapping population, associated with variation for flour colour [18]. Wheat *PSY*1 genomic sequences show similar exon-intron characteristic to *PSY*1 known in other Grass species, with six exons separated by five introns [18]. A partial sequence of *PSY*2 was also obtained and it was mapped on the short arm of the chromosome group 5 [13], with no clear data regarding the impact of *PSY*2 on carotenoid accumulation.

In this article, we report evidence that the wheat PSY gene family also consists of three paralogous genes and that *PSY*3 is characterized by a novel gene structure, consequence of intron loss events. Since the wheat *PSY*3 promoter region is characterized by *cis-*acting elements involved in plant response to abiotic stress, we studied changes in wheat PSY expression levels in leaves and roots during exogenous treatment with ABA. Finally, after comparative genomic investigation of PSY loci, we reconstructed the paleo-history for this gene family in Grasses identifying evolutionary events that have shaped PSY gene copies in modern species.

## Results

### PSY3 gene sequencing and mapping in bread wheat

**Figure 2:**
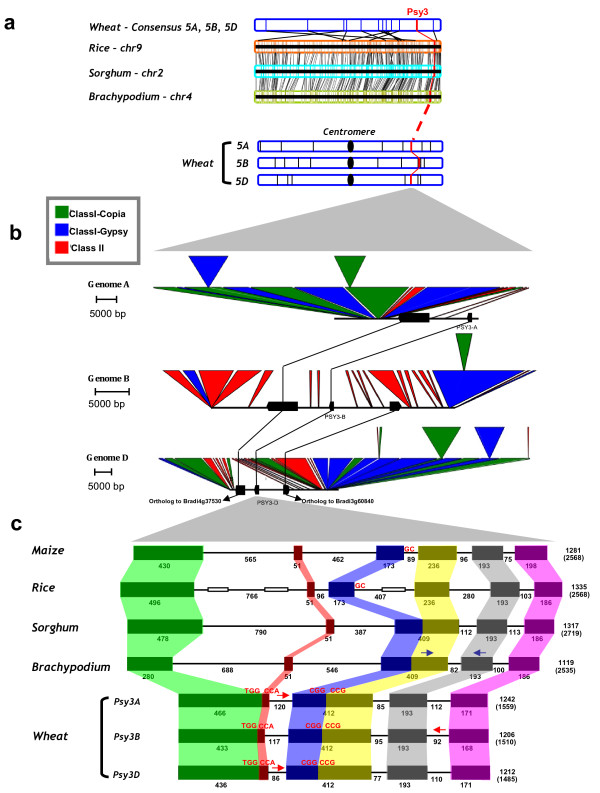
***PSY*3 gene characterization in bread wheat. (a)** The physical position of wheat *PSY*3 on chromosome group 5 and associated deletion bin are shown. Vertical bars on wheat chromosomes represent chromosome bins. The orthologous chromosomes in rice (Chr9), sorghum (Chr2) and *Brachypodium* (Chr4) are illustrated by conserved genes linked with black lines. **(b)** Annotated BAC clones are illustrated by conserved genes (black boxes) linked with black lines as well as Copia (in green), Gypsy (in blue) and class II (in red) transposable elements. **(c)** Comparison of *PSY*3 gene structures in Grasses (maize, rice, sorghum, *Brachypodium* and wheat) is shown based on coloured boxes highlighting conserved exons. Intron and exon sizes are shown as well as the total gene (in brackets) and CDS sizes. Maize and rice *PSY*3 share the same structure with six exons of conserved sizes and five introns. Their third intron shows a difference in size but in both rice and maize its 5′ starts with the same GC motif (highlighted in red), instead of GT. Sorghum and *Brachypodium PSY*3 show a structure of five exons and four introns. Wheat *PSY*3 have lost two introns probably due to the illegitimate recombination between the inversed repeated motives, TGG|CCA and CGG|CCG, identified at the deletion breakpoints (highlighted in red). White boxes represent MITEs identified on rice *PSY*3 sequence. Blue arrows show the position of primers designed on *Brachypodium PSY*3 sequence and used for BAC library screening. Red arrows correspond to specific primers, designed on intron sequence and used to assign BAC clones to A, B and D sub-genomes in wheat

We recently reassessed the syntenic relationships within monocots (rice, maize, *Brachypodium*, sorghum and Triticeae) and developed tools to identify precisely chromosome-to-chromosome orthologous relationships and derived Conserved Orthologous Set (COS) markers, [27,28]. Rice (LOC_Os09g38320), sorghum (Sb02g032370) and *Brachypodium* (Bradi4g37520.1) *PSY*3 orthologs, available from the literature, respectively mapped on chromosomes 9, 2 and 4 were used to clone wheat PSY genes. On the basis of the known rice/sorghum/wheat/*Brachypodium* syntenic blocks covering *PSY*3 orthologs, *PSY*3 was expected to be located on chromosome group 5 in wheat (Figure 2a, [29]). The blast search did not result in significant orthologous wheat sequences from public databanks (either EST database, [30] or the genome sequence database [31]). Primers were therefore developed from conserved genic regions characterized between rice and *Brachypodium PSY*3 coding sequences to screen a bread wheat BAC library (*T. aestivum cultivar* ‘Renan’ [32], see Materials and Methods). This library is organised in 384-well plates of BAC pools with a specific screening strategy to reduce the number of PCR reactions necessary to identify a single clone of interest (see Materials). Primer pairs PSY_4g37520.1_bra_F2/PSY_437520.1_bra_R2, designed on the fourth exon of *Brachypodium PSY*3 (Additional file 1: Table S1) were used for library screening. Positive pools were sequenced and three homoeoforms were obtained and assigned to the wheat chromosomes using nullisomic-tetrasomic lines [33]. By crossing the pool coordinates (plates/lines/columns) we identified the BAC clones harbouring *PSY*3 gene homoeologs. Three BAC clones spanning the *PSY*3 gene of A, B and D sub-genomes of *T. aestivum* were screened with PCR-specific markers targeting each of the three *PSY*3 homoeologs ( Additional file 1: Table S1 and Figure 2b–c) and then sequenced. Sequencing resulted in 138 391, 149 199 and 130 149 bp of gapped contigs for, respectively, the A, B and D sub-genomes. Each homoeologous copy was then precisely assigned to chromosome bins using genome-specific primer pairs ( Additional file 1: Table S1) and DNA from deletion lines: *PSY*3s were located precisely on the chromosome bins 5AL10-0.57-0.78, 5BL14-0.75-0.76 and 5DL1-0.60-0.74, respectively, for A, B and D genome copies.

### Identification and characterization of PSY3 loci

BAC sequencing and annotation (*cf*Materials and Methods) made it possible to identify *PSY*3 orthologous regions. We selected ungapped contigs of 25 000 bp, 53 411 bp and 46 803 bp harbouring, respectively, *PSY*3A, *PSY*3B and *PSY*3D genes ( Additional file 1: S1S2S3). Figure 2b illustrates BAC annotation results as well as micro-colinearity with rice, *Brachypodium*, maize and sorghum genome sequences, confirming that *PSY*3 and Bradi4g37530.1 genes are at orthologous positions. In the *PSY*3 loci we found the insertion of a non-syntenic gene (orthologous to Bradi3g60840). According to paleogenomic data in monocots [29], this gene is located on the syntenic chromosomes 2, 4 and 3 of, rice, sorghum and *Brachypodium* genomes respectively. In wheat, this gene would be expected to map on the orthologous chromosome group 6 and not group 5, where it was found based on the PSY sequenced region assignation. We can thus suppose that this gene has been specifically transposed in wheat from an ancestral donor region (group 6) to the modern current acceptor region (group 5).

**Figure 3:**
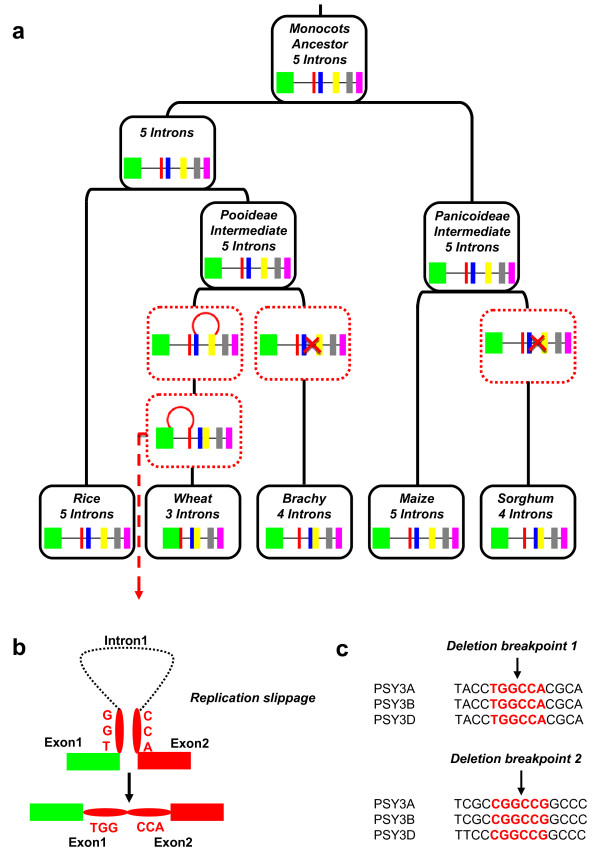
**Mechanism driving intron-exon shuffling of*PSY*3 genes in Grasses. (a)***PSY*3 gene evolution among Grasses. The modern structure of the *PSY*3 gene identified in rice, wheat, *Brachypodium*, maize and sorghum is shown at the bottom with coloured boxes representing conserved exons. The ancestral as well as pooideae and panicoideae *PSY*3 is structured in five introns and lineage-specific intron losses and consequently exon fusions in wheat, *Brachypodium* and sorghum are illustrated according to the text description. **(b)** Representation of intron loss mechanism identified for the wheat *PSY*3 with inverted and repeated motives that may have driven intron loss through replication slippage via the formation and a DNA loop. **(c)** Motifs identified at the deletion breakpoints and involved in intron loss due to illegitimate recombination at the splicing site are shown in red.

Alignment with *Brachypodium PSY*3 CDS clarified wheat *PSY*3s exon-intron boundaries. Wheat *PSY*3s showed a structure characterized by four exons spaced by three introns that were numbered according to their position within the genes, with the first intron closest to the 5′ and the third intron closest to the 3′ end (Figure 2c). This structure and exon-intron sizes were conserved between the A, B and D homoeologous copies. Rice and maize *PSY*3 were the only genes with six exons spaced by five introns. In *Brachypodium* and sorghum, the loss of the third intron determined the appearance of a different structure. In rice and maize, this intron showed at its donor site, the T/C transition, starting with the GC motif instead of GT. We also found in rice *PSY*3 the presence of Miniature Inverted Transposable Elements (MITEs), in the first intron: nt628-nt741 and nt953-nt1130, and in the third intron (nt1769-nt1955) the same MITE was identified in inverted orientation. Wheat *PSY*3 was found to include the largest first exon of 466 bp, 433 bp and 436 bp, respectively, for *PSY*3A, B and D, resulting from fusion of the first and second exons after an intron loss event. The second exon had 412 bp and resulted from the fusion of the third and fourth exons. It was characterized by a close sequence homology with the third exon of the *Brachypodium* ortholog. Figure 3a shows *PSY*3 gene structure evolution throughout Grasses. Rice and maize share the same *PSY*3 gene structure, while sorghum and *Brachypodium* gene structures are characterized, as in wheat, by the loss of the third intron and the subsequent fusion of the two flanking exons. We suggest that, during evolution, different and independent intron loss events occurred. At the deletion breakpoints, we identified repeated inversed motifs, TGG|CCA and CGG|CCG (Figure 3b, c) in the first and the second exon respectively. It is therefore possible to assume that these motifs have facilitated intron loss events through illegitimate recombination. The first intron lost was at phase 1, while the second was at phase 0. The TSSP database (see materials) defined the promoter region of *PSY*3, around 2 Kb upstream from the start codon and PolyA Signal Miner software identified the terminator signal 865 bp, 867 bp and 828 bp, respectively for A, B and D genomes. Sequence analysis allowed us to identify a duplication of the fourth exon in the *PSY*3D locus, 1729 bp downstream from the stop codon. This repeat did not start with the trinucleotide GTG, as expected based on the exon #4 sequence (but with the complementary nucleotides CAC), and was interrupted by a 154 bp deletion flanked at each extremity by the TACTGG motif. We can thus suppose that a novel illegitimate recombination event mediated the duplication of the fourth exon and its insertion in the sequence.

Despite intron/exon shuffling events reported previously, it was possible to predict protein structure. *PSY*3A encodes a protein of 413 amino acids, longer than those coded by *PSY*3B and *PSY*3D homoeologs (401 and 403 amino acids, respectively) ( Additional file 1: S1S2S3). The NCBI Conserved Domain Search software allowed us to identify the *trans*-Isoprenyl Diphosphate Synthase (*Trans*-IPPS) domain which includes squalene and phytoene synthases. According to the previous domain characterization, wheat *PSY*3 was classed in the IPSS superfamily and Class I terpene cyclise. The conserved *trans*-IPPS domain was identified at positions: aa123-386, aa112-375 and aa113-376, respectively, for *PSY*3A, B and D copies. Protein motifs identified based on ScanProsite are available in Additional file 1: Figure S1. ChloroP1.1 [34] identified transit peptides with 56 and 57 residues for *PSY*3B and *PSY*3D, but only 6-residues for *PSY*3A and a signal peptide of two amino acids for *Brachypodium PSY*3. However, according to ProtComp 9.0 [35] prediction, wheat *PSY*3 may have a putative chloroplast localization. We also analyzed sequence homology between wheat *PSY*3 proteins: *PSY*3A and *PSY*3B showed 91%, *PSY*3A and *PSY*3D 90% and *PSY*3B and *PSY*3D 96% amino acid sequence identity.

### Evolutionary history of PSY gene family

**Figure 4:**
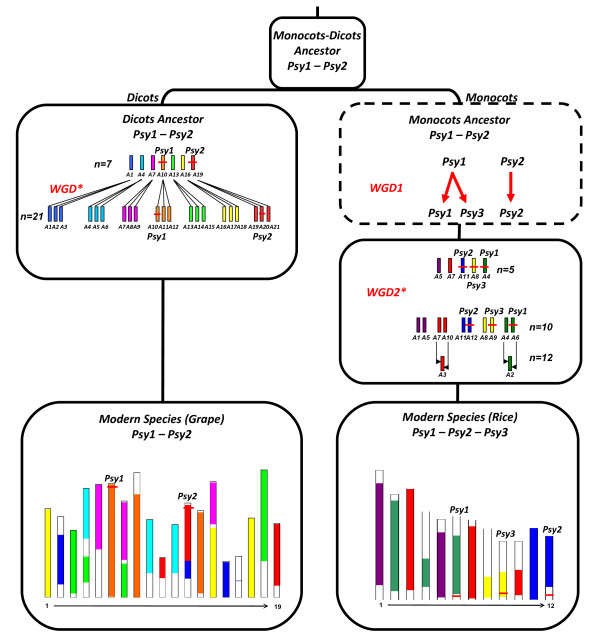
**PSY gene evolutionary scenario in Angiosperms.** The modern monocots (exemplified by the rice genome structured in 12 chromosomes) and eudicots (exemplified by the grape genome structured in 19 chromosomes) are illustrated at the bottom using a color code that reflect the ancestral genome structure of respectively 5 and 7 protochromosomes according to Abrouk *et al.*[28]. The angiosperm ancestor may contain two PSY gene copies, *i.e. PSY*1 and *PSY*2. The two copies are located on the protochromosomes A10 and A19 of the eudicot ancestor and both retained in single copies during the WGD shared by the eudicots. In modern eudicots, the PSY gene family is structured in two copies, *i.e. PSY*1 and *PSY*2. In monocots *PSY*1 has been duplicated during a first WGD (referenced as 1) so that the monocot ancestral genome contains three PSY gene copies, *i.e. PSY*1, *PSY*2 and *PSY*3. The three PSY genes are located on ancestral chromosomes A4, A11 and A8 and all retained in single copies during the WGD shared by the monocots (referenced as 2). In modern monocots, the PSY gene family is structured in three copies, *i.e. PSY*1, *PSY*2 and *PSY*3.

Throughout monocots, the PSY gene family is characterized by three paralogous genes, annotated as *PSY*1, *PSY*2 and *PSY*3, while, in eudicots, the presence of *PSY*1 and *PSY*2 homologs have been reported [5]. The presence of only two copies in eudicots was our starting point to explore evolution of the PSY gene family to explain the presence of a third copy in monocots. We proposed that the angiosperm ancestor was characterized by the presence of the two PSY genes, *PSY*1 and *PSY*2 (identified in monocots such as rice as well as in eudicots such as grape referenced in Figure 4), and a specific evolutionary event must have occurred in the monocots, duplicating *PSY*1 or *PSY*2 to give third copy *PSY3*. To test this hypothesis, we analyzed homology between the three PSY genes of*. Brachypodium*, rice and sorghum and observed that *PSY*1 was most closely related to *PSY*3 in terms of sequence similarity (>70% sequence identity at the nucleotide level). However, according to the precise characterization of duplicated chromosomes in monocots reported by Murat *et al.*[29], we concluded that *PSY*1-3 genes are not located on chromosomes that have been duplicated during the ancestral shared Whole Genome Duplication (WGD) dating back to 50–70 mya. *PSY*3 probably originated as a duplication of *PSY*1 during a more ancient and specific WGD event in monocot evolution that determined its modern localization in monocot genomes (Figure 4). This duplicated region corresponds to a more ancient WGD (≈130 million years old) that predate the 50–70 million years old ancestral paleotetraploidization in monocots [36,37].

### PSY genes expression profile and function investigation in bread wheat

Since there is no evidence of *PSY*3 expression in the literature, we used the public wheat Affymetrix chip data [38] based on array hybridization with RNA extracted from wheat grains at different development stages (100 Degree Days (DD), 200DD, 250DD, 300DD, 500DD), from cellular division to starch filling, to unravel the expression of PSY genes in wheat. We blast-searched the *PSY*3 sequences obtained against Affymetrix chip sequences and found two probes, Ta.20776.1.S1_at and Ta.18880.1.S1_at, homoeologs of *PSY*1 and *PSY*2, respectively. The expression level of *PSY*1 in grains was twice that of *PSY*2 during all developmental stages except at 500DD when *PSY*1 levels decreased ( Additional file 1: Figure S2). *PSY*2 expression was constant during grain development, whereas *PSY*1 expression exhibited significant differences between 350DD and 500DD (p-value = 2.22E-10, using the Bonferroni test). Our data showed that *PSY*1 increased at 200DD while its level remained constant at 300DD and then decreased at 500DD. No evidence was found for *PSY*3 expression in grain at these developmental stages. To gain insight into PSY gene expression during grain development, we used Single-Strand Conformation Polymorphism (SSCP) analysis [27] to screen wheat RNAs (*cv* Recital) extracted from leaves, roots and grains at different development stages. SSCP offered in this experiment the opportunity to (i) detect minor sequence changes in polymerase chain reaction-amplified samples and (ii) evaluate the expression level of three homoeologous copies of each PSY gene in the same running time [38]. In the same PCR reaction, we amplified specifically one paralogous gene and its homoeologous copies that were successfully separated, by capillary electrophoresis according to their sequence differences and assigned to each genome, using amplification profiling approaches on nullitetrasomic lines as references [38]. *PSY*1 expression was evaluated based on the primer pair *PSY*1_F1R1 (amplification product of 218 bp) designed on the fourth exon. Its expression in developing grain (100DD, 200DD, 250DD, 300DD and 500DD) was constant and *PSY*1-B showed the highest transcript level. *PSY*1 expression profile did not change in leaves and roots where *PSY*1-B was always the copy most expressed. We also amplified *PSY*2 in each tissue using the primer pair *PSY*2F1R1, designed on the fourth exon (amplification product of 141 bp). *PSY*2 showed a constant expression pattern in developing grains, the B and D copies showing the highest transcript levels. In leaves, all three homoeolog copies presented the same expression profiles; whereas, in roots, the D transcript decreased compared to B and A, whose levels remain unchanged. Using this SSCP approach we were able to detect *PSY*3 expression using the primer pair *PSY*3F2R1, designed on the third exon (amplification product of 216 bp). During grain development at 100DD the D copy showed the highest transcript level, while, at 200DD, amplification intensity of the B copy increased in contrast with the two other homoeologs, which decreased. For the remaining stages, expression levels of the three homoeologous copies were constant. In leaves, A and D copies had the highest expression levels, while in roots, the D copy showed a greater expression than the A and B copies ( Additional file 1: Figure S3).

**Figure 5:**
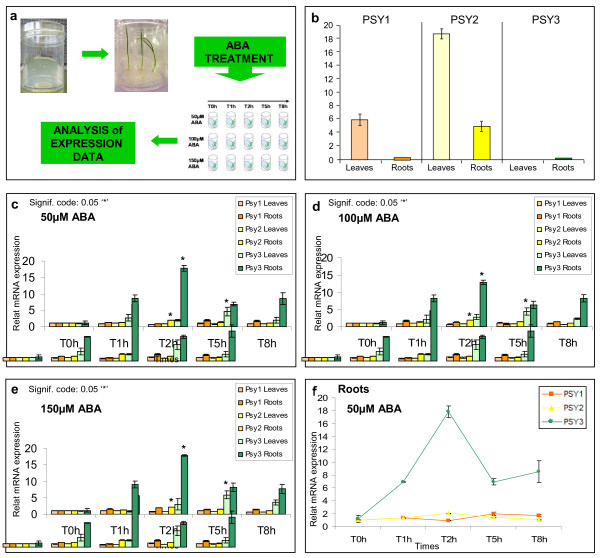
**Effect of ABA treatment on wheat PSY expression. (a)** Experimental design. **(b)** Expression level of PSYs in non-stressed leaves and roots. Transcript levels were normalized using the wheat spastin and RNase L inhibitor-like cDNA. **(c) (d) (e)** Figures show PSY response (y-axis) to ABA treatments at the three different tested concentrations (50 μM in c panel, 100 μM in d panel and 150 μM in e panel) in both leaves and roots (see legend) from 0 to 8 hours (x-axis). cDNA and expressed relative to the level detected in not stressed tissues. Each stage has been compared two by two with a confidence level of 95%. The statistical significance in gene expression is shown by an astrisck (*). **(f)** Efficiency of ABA on *PSY*3 induction in wheat roots.

We exploited the wheat *PSY*3 promoter sequence to search for regulatory elements. Toucan 9.0 workbench software revealed the presence of conserved *cis-*regulatory elements such as, ABA-responsive element (ABRE), G-box and a Coupling Element (CE), involved in the response to salt and drought stress and application of ABA, and also identified in rice and sorghum *PSY*3 promoters [11,39]. In order to confirm this hypothesis, Real-Time PCR was used to investigate the expression levels of all three PSYs in leaves and roots during the application of exogenous abscisic acid (ABA). Three concentrations of ABA (50 μM, 100 μM and 150 μM) were tested and samples from each tissue were collected after 0, 1, 2, 5 and 8 hours (h) of treatment (Figure 5a). In photosynthetically non-stressed tissues, *PSY*1 and *PSY*2 gave the major transcripts, which were three times greater from *PSY*2 than from *PSY*1 (Figure 5b). In non-stressed roots, *PSY*1 expression was low and *PSY*2 expression levels were at least more than 3 times lower than in leaves (Figure 5b). This result agrees with the hypothesis that, in wheat, *PSY*1 is directly involved in carotenoid accumulation in grain, associated with major QTLs for YP [13,18,24] whereas *PSY*2 plays a major role in carotenoids accumulation in leaves.

To investigate changes in PSY expression during stress, we determined transcript levels in leaf and root samples after stress treatment. PSY responses to the different ABA concentrations tested were constant. *PSY*1 mRNA levels, in stressed leaves, underwent to a minimum reduction during the first 2 h to then returned to the initial level at 5 h. *PSY*2 transcripts slightly decreased in stressed leaves after 2 h and then remained constant. *PSY*3 expression levels increased about 4-fold after 5 h of stress treatment and then returned to initial levels. In stressed roots, differences in expression levels became more obvious: *PSY*1 mRNA levels doubled after 5 h of treatment, while *PSY*2 transcript levels doubled after only 2 h of stress and then decreased gradually after 5 and 8 h of treatments to initial expression levels (Figure 5c-d-e). *PSY*3 expression levels started to increase just 1 h after the ABA application to 18-fold after 2 h and then returned to lower levels (see Additional file 1: Table S2 for ANOVA analysis). Overall, Figure 5f clearly shows the effectiveness of ABA treatment on *PSY*3 up-regulation in roots. Normalized expression data of PSY genes obtained in this experiment are available as Additional file 1: Figure S4.

## Discussion

Carotenoids are widely recognized as essential pigments in plants because of their role in development, photosynthesis, photoprotection and in response to stress since they are essential precursors of apocarotenoids, such as the abscisic acid (ABA). The PSY gene family, playing a central role in carotenoid biosynthesis, is characterized by duplicated gene copies. Their characterization in Grasses and the recent identification of a third copy in rice, maize and sorghum raises questions about PSY gene family organization in wheat. The role and function of *PSY*1 have been largely investigated because of its association with major QTLs for carotenoid accumulation in wheat grain. Partial sequences of *PSY*2 from several wheat varieties are available but little is known about its function and, up to now, there was no evidence of a *PSY*3 homolog and associated activity in wheat. Our current analysis has demonstrated that wheat PSY gene family is characterized by the existence of *PSY*1 to 3 gene copies. We report here the precise nature of gene family structure, evolution and function.

Because of the polyploid nature of wheat and the lack of a whole genome sequence, we identified orthologous regions in reference genomes such as *Brachypodium* and developed COS markers to isolate wheat *PSY*3 from a BAC library. Wheat *PSY*3 is characterized by a novel gene structure due to intron loss events leading to four exons and three introns. Rice and maize *PSY*3, whose gene structure is highly conserved, are characterized by a third intron starting with a non-canonical 5′splicing site GC instead of GT. This type of intron is deleted by the same spliceosome mechanism that removes GT-AG introns, mainly involved in alternative splicing rather than in introns loss events [40]. *Brachypodium* and sorghum show differences in PSY gene structure, characterized by five exons and four introns. The third intron has been deleted but the causal mechanism is not yet clear. The classical model to explain intron loss is based on recombination of a genomic copy of a gene with a reverse-transcribed copy of a spliced mRNA transcript, deleting one or more adjacent introns. A key aspect of this theory is that only multicellular eukaryotes that have genes transcribed in germ cells would be susceptible to mRNA-mediated intron loss [41-45]. We identified inverted and repeated short motifs at deletion breakpoints in the wheat *PSY*3 sequence, corresponding to the 5′ and the 3′ splicing sites, which may have driven illegitimate recombination leading to the observed intron loss events. *Brachypodium* and sorghum *PSY*3 sequences are not characterized by such repeated motifs at deletion breakpoint, suggesting that intron loss occurred by simple genomic deletion. Maize PSY3 intron 2 and rice intron 3 and maize intron 3 and rice intron 2 are similar in size, leading to the hypothesis that these introns may have been inverted but this could not be confirmed due to limited sequence similarities between introns. Howitt *et al.*[46] described the presence within the intron #3 in wheat *PSY*-D1 of an insertion of 1200 bp of an inverted repeat motif. The characterization of either repeated motifs and/or TEs in this intron in Grasses may have driven intron loss or shuffling events leading to the distinct *PSY*3 gene structures observed.

PSY duplicated genes exist throughout monocots and eudicots. Gallagher *et al.*[5] proposed an evolutionary model for the Grass PSY gene family in which monocots and eudicots share the same ancestral gene and the same duplication event, giving rise to genes *PSY*1 and *PSY*2. Welsch and collaborators [11] proposed that rice *PSY*3 originated from *PSY*2 duplication or *vice versa*, because of their close similarity in the 5′UTR region. We analyzed homology between PSY genes in reference genomes and showed that *PSY*3 had greater homology with *PSY*1 sequence than *PSY*2, suggesting that *PSY*3 originated from a duplication of *PSY*1. Furthermore, public paleogenomics data in Grasses indicated that PSY genes are not located on known duplicated chromosomes originating from the characterized ancestral WGD event reported by Murat *et al.*[29]. We suggest, according to Jaillon *et al.* and Tang *et al.*[36,37], that a more ancient WGD event, specific to monocots has shaped the *PSY*3 copy as a duplication of *PSY*1.

Based on a semi-quantitative approach, such as SSCP strategy, we observed PSY expression in wheat grains, leaves and roots. *PSY*1 and *PSY*2 homoeologous copies showed stable expression profiles in the different tissues. *PSY*-B1 was the copy most expressed, while, *PSY*-B2 and *PSY*-D2 showed the largest transcript levels in developing grains and, in leaves and roots, *PSY*-A2 transcripts increased. *PSY*3 homoeologous copies showed a variable pattern of expression during the first stage of cellular division during early grain development and we found *PSY*-A3 and *PSY*-D3 most expressed in leaves and *PSY*-D3 in roots. However, with this approach, a statistically basis comparison was not possible between the expression levels of the three paralogous genes because it is semi-quantitative and only the broad transcript levels of homoeologous copies of each gene can be compared when amplified with the same primer pairs and in the same conditions. Access to recently published Affymetrix data [38] provided the opportunity to investigate PSY gene expression pattern and showed that *PSY*1 expression level was higher than *PSY*2 in developing grains. *PSY*1 transcripts increased at 200DD to remain constant until 500DD, when they were reduced. In developing maize endosperm, Li *et al.*[12] showed that during endosperm carotenogenesis, only *PSY*1 transcript increased significantly, starting after 12DAP until 22DAP. Basis on this evidence, we can speculate that wheat *PSY*1 in the endosperm follows the same kinetics as the maize *PSY*1 ortholog and that apparently there is a correspondence in timing of expression between wheat and maize *PSY*1 orthologs. Our results are consistent with the evidence that *PSY*1 plays an important role in carotenoid accumulation in the endosperm and with its association with QTLs for YP on chromosome group 7 in wheat [13,18,24].

Gene expression is closely associated with *cis-*regulatory DNA sequences (known as *cis*-elements) located in its upstream regions [47]. In rice and maize, *PSY*3 expression is suggested to be strongly inducible in roots by drought and salt stress and application of ABA, since their promoter regions harbour *cis*-elements involved in plant response to abiotic stress [11,39]. We identified several regulatory elements in the wheat *PSY*3 promoter region, (*i*) the ABA-responsive element (ABRE) whose core is represented by the G-box, and (*ii*) a Coupling Element (CE1 or CE3) that is necessary to obtain ABRE functional [48,49]. Through a Real-Time PCR approach, we quantified wheat PSY transcript levels in leaves and roots before and after the exogenous application of ABA, observing that *PSY*2 represents the most abundant transcript in unstressed leaves, while *PSY*1 transcript levels were 3-fold lower than in carotenoid accumulation in grains [13,18,24]. Wheat *PSY*2 may thus play an important and complementary role in YP accumulation in photosynthetic tissues. *PSY*3 expression levels were low in non-stressed leaves compared to *PSY*1 and *PSY*2 while its levels increased in non-stressed roots. *PSY*1 and *PSY*2 transcript levels did not show significant changes in leaves after ABA treatment. In stressed roots, *PSY*1 and *PSY*2 levels doubled after 5 h and 2 h of treatment respectively. *PSY*3 showed the most intense response to ABA as in leaves, after 5 h of ABA application, *PSY*3 transcript was 4-fold higher, while in stressed roots after 2 h its transcript levels increase about 18-fold. *PSY*3 was the only gene that showed significant changes of transcript levels in the studied conditions. Carotenoids provide precursors for ABA which is the main apocarotenoid that plants can accumulate under drought conditions, ABA applications having been shown to affect plant growth and development, mimicking the effect of water stress and improving capacity to escape or tolerate such stress [50]. Since PSY genes are considered as rate-limiting in carotenogenesis, we can explain the up-regulation of *PSY*3 by ABA as the necessity of plants to control carotenoid flux to roots during stress conditions to provide ABA precursors and thus activate stress responses. In a previous study, Singh *et al.*[15], studying the association of *PSY*1-A1 allelic variation with YP colour in durum wheat, speculated about the possible existence of a third PSY on the chromosome 7AL to explain the presence of a second QTL near the marker *Xwmc116*. We identified the chromosomal bin localization for each homoeolog and the expected syntenic relationships were confirmed assigning *PSY*3 to chromosome group 5 which has the highest concentration of QTLs and major loci controlling plant adaptation to environment, particularly, those controlling heading date, frost and salt tolerance, whereas a region with a crucial role in drought tolerance is located on chromosome group 7. Multiple-stress QTLs and linked markers have also been detected, suggesting the existence of common mechanisms for different stresses or of gene clusters controlling different stress tolerance processes [51]. For example, in wheat a significant QTL for ABA accumulation in droughted leaves was found on chromosome 5A co-located with the major vernalization responsive gene, Vrn1 [50], and in the same chromosomal bin we mapped *PSY*-3A. Aprile *et al.*[52] studied different physiological reactions to water stress and the substantially different molecular responses of bread and durum wheat genotypes demonstrating that there is a difference between durum and bread wheat response to water stress and that deletion of bin 5AL-10 from Chinese Spring determined a lower response to such stresses. They also showed that the different response by durum and bread wheat can be associated with the absence of D genome, where genes or factors that modulate expression of cluster of genes may be localized. *PSY*3 localization and function in wheat are consistent with these data, especially the *PSY*3D genomic structure, function and expression reported in the current study. Finally, we also showed here that *PSY*3s are characterized by intron losses, inverted repeats and up-regulation by abiotic stresses. It is important to note that biotic/abiotic stresses could also activate transposons and retrotransposons [53] whose expression may have played a role in *PSY*3 gene structure modification.

## Conclusion

PSY gene family plays an important role in plant development (photoprotection and resistance to stress) as well as is a key contributor to flower and fruit flavours and aromas. The current analysis provides relevant structural (sequence, primers), functional (expression patterns) and evolutionary (gene conservation and specificity) informations that can be used as applied tools in wheat breeding programs.

## Methods

### PSY gene orthologs identification

The methodology used to reassess synteny between wheat/rice/*Brachypodium*/sorghum/maize genomes as well as the identification of intra-chromosomal duplications in wheat was previously described [28,29,54,55]. Briefly, wheat [30,31], rice (41046 genes), *Brachypodium* (25504 genes), sorghum (34008 genes) and maize (32540 genes) genomes were aligned with the publicly available PSY gene sequence [56] to identify orthologs. Three parameters were used to increase stringency and significance of BLAST sequence alignment by parsing BLASTN results and rebuilding HSPs (high scoring pairs) or pairwise sequence alignments. The first parameter, AL (aligned length), corresponds to the sum of all HSP lengths. The second, CIP (cumulative identity percentage) corresponds to the cumulative percent of sequence identity obtained for all the HSPs (CIP = ∑ nb ID by HSP/AL) x 100). The third parameter, CALP is the cumulative alignment length percentage. It represents the sum of the HSP lengths (AL) for all the HSPs divided by the length of the query sequence (CALP = AL/Query length). These parameters allow identification of the best alignment, with the highest cumulative percentage of identity in the longest cumulative length, taking into account all HSPs obtained for any pairwise alignment, regarding PSY sequences. These parameters were applied to all the BLAST alignments that were performed in the present study. Based on the genome-wide synteny analysis, gene relationships between species were then referenced as COS (for conserved gene pairs), CNV (for tandem duplicated genes) and PAV (for non –conserved genes).

### BAC clone screening, sequencing and annotation

A BAC library from *T. aestivum* ‘Renan’ [32] was used in this study, the library has a 6.9x genome coverage. It consists of 812,544 clones organized in 2592 384-well plates pooled in two steps. The first step aims at mixing the 384 clones of each plate; the second step organizes plate pools in two dimensional matrices. For each 384-well plate, 16-line pools and 24-column pools and a total of 40 pools were available. Overall, 274 line and column pools were available to identify BAC clones of interest through PCR screening using PSY primers. Each amplicon (or BAC of interest) in a 384-wells plate was identified by coordinates which identified positive line and column pools in a matrix. By crossing the coordinates of positive pools we determined the plate harbouring the clone of interest. The individual BAC clone of interest was then identified on specific plates by PCR screening. BAC sequencing was performed on the GS Junior (Roche) using the 454 sequencing technology according the procedure described by the manufacturer at the genotyping platform GENTYANE (UMR-1095 GDEC, Clermont-Ferrand).

Genes and repeated elements (TEs) were identified by computing and integrating results based on BLAST algorithms [57], predictor programs and software described below. Gene structure and putative functions were identified by combining results of BLASTN and BLASTX alignments against dbEST [58] and SwissProt databases [59] with the results of predictor program, FgeneSH [60] with default parameters. Known genes were named based on BLASTX results against protein with known functions (SwissProt). Promoter analysis was performed using both TSSP database and Toucan platform [61] to search for *cis*-elements. PolyA Miner Signal [62] was used in terminator analysis. Characterization of protein functional domain was performed using InterPro [63] and SMART database [64,65], while protein motifs were analysed with ScanProsite [66]. ChloroP1.1 and ProtComp 9.0 were used, respectively, to identify signal peptide and to predict protein subcellular localization.

Transposable elements (TEs) were detected by comparison with two databases of repetitive elements: TREP [67] and Repbase [68]. TEs boundaries were identified with the REPET package [69]. Insertion profile of TEs is identified using a modified version of svg_ltr.pl script [70].

### Stress treatment experimental design and quantitative real-time PCR

Recital seeds were surface sterilized and embryos at 16 days after pollination (DAP) were collected and grown on Murashige and Skoog agar medium with 2% saccharose and indole-3-acetic acid (IAA). In each pot, three embryos were sown in order to obtain three biological replications for each condition. Plants were grown during the first 3 days in the dark at 25 °C and were transferred to continuous light at 22 °C at the two-leaf stage.

To carry out stress experiments, plants were treated with solution of ABA at different concentration: 50 μM, 100 μM and 150 μM. Leaf and root samples were collected after 0, 1, 2, 5 and 8 hours (h) of ABA treatment and were stored at −80 °C. RNA extraction was performed according to TRIzol® Reagent protocol. DNase set (Qiagen) and RNeasy Minelute Cleanup (Qiagen) kits were used for RNA purification. Integrity and quality of mRNA (contamination assessment of genomic DNA) were checked with the A_260_/A_280_ ratio with Nanodrop (Thermo Scientific) and on agarose with a classical PCR using a reference gene (see primers below). cDNA synthesis was carried out with a Transcriptor first strand cDNA synthesis kit (Roche) following the procedure described by the manufacturer with 1 μg of RNA (cycling conditions: 10′ at 25 °C/30′ at 55 °C/5′ at 85 °C°). Four primer pairs used for Real-Time PCR analysis ( Additional file 1: Table S1) were designed with primer3 in order to obtain exonic specific amplification of 100pb. Real-time PCR was performed using the LightCycler® 480 (Roche Diagnostics) and all reactions were performed using the LightCycler® 480 DNA SYBR Green I Master (Roche Diagnostics) in 10 μl with 4 ng of cDNA and 55 PCR cycles at 60 °C (hybridation temperature). All samples were prepared in three technical replicates and a negative control using water as a template was included. Quantification cycles (Cq) were analysed using the Light Cycler software version 1.5.0 and normalized with two reference genes (wheat spastin (primers: TGCCACTGCCTGATCCAAAT and AGCAAGCCTCTCCAGATCATG) and RNase L inhibitor-like (primers: TTGAGCAACTCATGGACCAG and GCTTTCCAAGGCACAAACAT) [71]) using ‘Advanced Relative Quantification’ module to obtain normalized ratio E_t_^-CqT^/E_r_^-CqR^ (with CqT/CqR : Cycle number at target/reference detection threshold (crossing point) and E_T_/E_R_ : Efficiency of target/reference amplification (10^-1/slope^)). Specificity of amplification was confirmed via melting curve analysis of final PCR products by increasing the temperature from 65 °C to 95 °C. PCR efficiency was calculated for each gene using a standard curve of serial dilutions and used in relative expression analysis. To calculate transcript abundance under stress conditions, the transcript levels were obtained for the controls, at each considered time point and ABA concentration and tissues. All observations were expressed as mean ± SD; the significance (*P* ≤ 0.05) of variables studied was assessed by one-way analysis of variance (ANOVA) using R software version 2.13.1.

## Competing interests

The authors declare that there are no competing interests.

## Authors’ contribution

BD designed the experiment, performed the analysis and participated in manuscript preparation. FM performed the bioinfomatic analysis and participated in manuscript preparation. CP, AC, VG, CP, IM, HB contributed to the BAC clone screening, sequencing and annotation. CP and PL contributed to the expression analysis. JS and AB designed the research program. JS managed the research group and wrote the article. All authors read and approved the final manuscript.

## Supplementary Material

Additional file 1 **S1.***PSY*3A gene sequences. **S2.***PSY*3B gene sequences. **S3.***PSY*3D gene sequences. **Table S1.***PSY*3 gene primer sets. **Table S2.** p-value from one-way ANOVA analysis using R software. **Figure S1.***PSY*3 homoeo-alleles protein sequences. **Figure S2.***PSY*1 and *PSY*2 expression profile in developing grains. **Figure S3.***PSY*3 homoeo-alleles expression during grain development. **Figure S4.** PSYs expression after ABA treatment. Click here for file
