# Supplementary material for: Deciphering the genomic structure, function and evolution of carotenogenesis related phytoene synthases in grasses
Source: BMC Genomics. 2012 Jun 6;13:221. doi: 10.1186/1471-2164-13-221 (PMC3413518; doi:10.1186/1471-2164-13-221)

**Supplemental File S1: *PSY3A gene sequences***

The gene (PSY3A_Gene), CDS (PSY3A_CDS), protein (PSY3A_Protein) sequences are provided as classical FASTA files.

**>PSY3A_Gene**

ATGCTCTCCACGGCCCGCGCGGTGACCTCGCCCGCCGCGTGCGCTGCCCAGCACCAGCAGCATGGCGGGAGCCGGTTCGTCGTGTTCCTGGCCGGCGATGACGGGAGGGGAGCTGCCGGTTCCAGCAGCGTGCGTGCGCCGAGGCCGCGCTCTGTGGCAGCGCGTGCCGCCGGCTCGTCCAACCACGCTGTCTGCCTCGAGGCGGCCGCGCCGTGGAGCATGGACTGTGTCGCGGCGCCGGCGACCGTGTCGTACGAGGAGAGCGTGCGCCAGGTCGTGCTCAAGCAGGCGGCGCTCGCGGCCTCGACCACGCCGAGGAAGGCGCGGCACGCCGGCCGTGACGCCGGCGTGCTGGACGCGGCCTTCGACCGCTGCGGCGCGGTGTGCAAGGAGTACGCCAAGACGTTCTACCTGGCCACGCAGCTGATGACGCCGGAACGGAGGCGGGCGATCTGGGCGATATACGGTAAGCACTCGTCGATCGCGACATGCTGCCACTAGCTTCCACGTTGGTGGACGCAACGCAAGTACGCAACAGCAGGAGGTCATTGATGTGGTTAACTTCTTGGATGCGTCGCGCATGCAGTGTGGTGCCGGAGGACGGACGAGCTGGTGGACGGCCCGAACGCGGCGCACACGTCGGCGCTGGCGCTGGACCGCTGGGAGTCGCGCCTCGACGGCGTCTTCGCCGGCCGGCCCTACGACATGCTCGACGCGGCGCTGGCCGACGCCGTGGCCGCCTTCCCGGCGGTCGACGAGCGGCCGTTCCGGGACATGGTCCAGGGGATGCGCATGGACCTCGCCAAGTCCCGCTACGCCACCTTCGACGAGCTCTACCTCTACTGCTACCGCGTGGCCGGCACCGTGGGGCTCATGACCGTCCCCGTCATGGGTGTCTCCCCGGGCTCCCAGGCGGACGTGGAGACGGTGTACGCCGGCGCGCTCGCCCTCGGCGTCGCCAACCAGCTCACCAACATCCTCAGGGACGTCGGCGAGGAGTAAGTGTCTGTATAGTATGTTGCTTCTGAATTCACGCGCTCTTGCCGATGACTGCTCATCTCTGACACCGCCATTGTTGCACAGTGCGAGGAGGGGAAGGATCTACCTGCCGCAGGACGAGCTAGCCATGGCCGGCATCTCCGAGGCGGACATCTTCGCCGGCCGTGTCACCGACGAGTGGAGGAGCTTCATGAAGGGACAGATCGCGAGGGCCAGGGCCTACTTCAAACAAGCCGAGCAAGGCGCCGCCGAGCTCAACCAGGAGAGCCGATGGCCGGTACGCGCGCGACAAAAGTTCCATCAAAAAATTCAAAAATTCTCAGCCAAAAATGTCAAATTCAGTAGTCTAAGTAAGTTTCAGATTAACATGTAATACGATTTGCATGCAGGTGTGGGCATCTCTGCTTCTGTACCGGCAGATCCTGGACGAGATCGAGGCCAACGGCTACGACAACTTCAGCAAGCGGGCCTATGTTCCCAAGGCCAAGAAGCTGGCGGCGCTGCCCAAAGCTTACCTCAGATCCCTCATGCCCCCTCCTTCGCAGACGCGCCGTTAA

**>PSY3A_CDS**

ATGCTCTCCACGGCCCGCGCGGTGACCTCGCCCGCCGCGTGCGCTGCCCAGCACCAGCAGCATGGCGGGAGCCGGTTCGTCGTGTTCCTGGCCGGCGATGACGGGAGGGGAGCTGCCGGTTCCAGCAGCGTGCGTGCGCCGAGGCCGCGCTCTGTGGCAGCGCGTGCCGCCGGCTCGTCCAACCACGCTGTCTGCCTCGAGGCGGCCGCGCCGTGGAGCATGGACTGTGTCGCGGCGCCGGCGACCGTGTCGTACGAGGAGAGCGTGCGCCAGGTCGTGCTCAAGCAGGCGGCGCTCGCGGCCTCGACCACGCCGAGGAAGGCGCGGCACGCCGGCCGTGACGCCGGCGTGCTGGACGCGGCCTTCGACCGCTGCGGCGCGGTGTGCAAGGAGTACGCCAAGACGTTCTACCTGGCCACGCAGCTGATGACGCCGGAACGGAGGCGGGCGATCTGGGCGATATACGTGTGGTGCCGGAGGACGGACGAGCTGGTGGACGGCCCGAACGCGGCGCACACGTCGGCGCTGGCGCTGGACCGCTGGGAGTCGCGCCTCGACGGCGTCTTCGCCGGCCGGCCCTACGACATGCTCGACGCGGCGCTGGCCGACGCCGTGGCCGCCTTCCCGGCGGTCGACGAGCGGCCGTTCCGGGACATGGTCCAGGGGATGCGCATGGACCTCGCCAAGTCCCGCTACGCCACCTTCGACGAGCTCTACCTCTACTGCTACCGCGTGGCCGGCACCGTGGGGCTCATGACCGTCCCCGTCATGGGTGTCTCCCCGGGCTCCCAGGCGGACGTGGAGACGGTGTACGCCGGCGCGCTCGCCCTCGGCGTCGCCAACCAGCTCACCAACATCCTCAGGGACGTCGGCGAGGATGCGAGGAGGGGAAGGATCTACCTGCCGCAGGACGAGCTAGCCATGGCCGGCATCTCCGAGGCGGACATCTTCGCCGGCCGTGTCACCGACGAGTGGAGGAGCTTCATGAAGGGACAGATCGCGAGGGCCAGGGCCTACTTCAAACAAGCCGAGCAAGGCGCCGCCGAGCTCAACCAGGAGAGCCGATGGCCGGTGTGGGCATCTCTGCTTCTGTACCGGCAGATCCTGGACGAGATCGAGGCCAACGGCTACGACAACTTCAGCAAGCGGGCCTATGTTCCCAAGGCCAAGAAGCTGGCGGCGCTGCCCAAAGCTTACCTCAGATCCCTCATGCCCCCTCCTTCGCAGACGCGCCGTTAA

**>PSY3A_Protein**

MLSTARAVTSPAACAAQHQQHGGSRFVVFLAGDDGRGAAGSSSVRAPRPRSVAARAAGSSNHAVCLEAAAPWSMDCVAAPATVSYEESVRQVVLKQAALAASTTPRKARHAGRDAGVLDAAFDRCGAVCKEYAKTFYLATQLMTPERRRAIWAIYVWCRRTDELVDGPNAAHTSALALDRWESRLDGVFAGRPYDMLDAALADAVAAFPAVDERPFRDMVQGMRMDLAKSRYATFDELYLYCYRVAGTVGLMTVPVMGVSPGSQADVETVYAGALALGVANQLTNILRDVGEDARRGRIYLPQDELAMAGISEADIFAGRVTDEWRSFMKGQIARARAYFKQAEQGAAELNQESRWPVWASLLLYRQILDEIEANGYDNFSKRAYVPKAKKLAALPKAYLRSLMPPPSQTRR

**Supplemental File S2: *PSY3B gene sequences***

The gene (PSY3B_Gene), CDS (PSY3B_CDS), protein (PSY3B_Protein) sequences are provided as classical FASTA files.

**>*PSY*3B_Gene**

ATGCTCTCCACCGGCCGCGCGGTGACCTCGCCCGCGCGCGCTGCCCAGCACCGGTTCATGGGCGCACCGAGGACGGTGGTGTTCCTCGCCGGCGACCACGGGAAGCGAGCTGCTGCTTCTGTGGCAGCGCGCGCCGCGGGCTCGTCCCACCCCGCTGTCTGCCTCGAGGCGGCCGCGCCATGGAGCATGGACTGTGTCGCGGCGCCGGCGACCGTGTCGCACGAGGAGAGCGTGCGCCAGGTCGTGCTCAAGCAGGCGGCGCTCGCGGCCTCGACCACGCCGAGGAAGGCGCGGCTCGCCGGGCGCGACGCCGGCGTGCTGGACGCGGCCTTCGCGCGCTGCGGCGCGGTGTGCAAGGAGTACGCCAAGACCTTCTACCTGGCCACGCAGCTCATGACGCCGGAGAGACGGCGGGCGATCTGGGCGATATACGGTAAGGCCCCCCGTCGATCGCGACATGCTGCCACTAGCTAACTTCCACGCTGGTGGACGCAACGCAACAGCAGGAGGTCACAGATGTGGTTACTAACTTCTTGGATGCATGGCGCAGTGTGGTGCCGGAGGACGGACGAGCTGGTGGACGGCCCGAACGCGGCGCACACGTCGGCGCTGGCGCTGGACCGCTGGGAGTCGCGCCTCGACGGCGTCTTCGCCGGCCGGCCCTACGACATGCTGGACGCGGCGCTGGCCGACGCCGTGGCCGCCTTCCCGGCCGTCGACGAGCGGCCGTTCCGGGACATGGTCCAGGGCATGCGCATGGACCTCGCCAAGTCCCGCTACGCCACCTTCGACGAGCTCTACCTCTACTGCTACCGCGTGGCCGGCACCGTCGGGCTCATGACCGTCCCCGTCATGGGCGTCTCCCCGGGCTCCCAGGCGGACGTCGAGACGGTGTACGCCGGCGCGCTCGCCCTCGGCGTCGCCAACCAGCTCACCAACATCCTCAGGGACGTCGGCGAGGAGTAAGTGTCAGTTCAGTTGTATATATACCTCGCTTTCTGAATTCACCTGCTCTTGCCGCTGCCCGCTCATCTCTGACGCCGCCATTGTTGCACAGTGCGAGGAGGGGCAGGATCTACCTGCCGCAGAACGAGCTGGCCACGGCCGGCATCTCCGAGGCGGACATCTTCGCCGGCCGCGTCACCGACGAGTGGAGGAGCTTCATGAAGGGCCAGATCGCGAGGGCCAGGGCCTACTTCCAGCAGGCCGAGCAAGGCGCCGCCGAGCTCAACCAGGAGAGCCGATGGCCGGTAAGTGCGACGGACACTTACGCATGACACTTTCTTCTTCAATCACCTCGCAGAAATTCCATCAAAGATTAACATGTAATTTGTGCATGCAGGTGTGGGCATCTCTGCTTCTGTACCGTCAGATCCTGGACGAGATCGAGGCCAACGGCTACGACAACTTCAGCAAGCGGGCCTATGTTCCCAAGGCCAAGAAGCTGGCGGCGCTGCCCAAAGCTTACCTCAGATCCCTCATGCCCCCTCCTTCGCAGACGCGCCGTTAA

**>*PSY*3B_CDS**

ATGCTCTCCACCGGCCGCGCGGTGACCTCGCCCGCGCGCGCTGCCCAGCACCGGTTCATGGGCGCACCGAGGACGGTGGTGTTCCTCGCCGGCGACCACGGGAAGCGAGCTGCTGCTTCTGTGGCAGCGCGCGCCGCGGGCTCGTCCCACCCCGCTGTCTGCCTCGAGGCGGCCGCGCCATGGAGCATGGACTGTGTCGCGGCGCCGGCGACCGTGTCGCACGAGGAGAGCGTGCGCCAGGTCGTGCTCAAGCAGGCGGCGCTCGCGGCCTCGACCACGCCGAGGAAGGCGCGGCTCGCCGGGCGCGACGCCGGCGTGCTGGACGCGGCCTTCGCGCGCTGCGGCGCGGTGTGCAAGGAGTACGCCAAGACCTTCTACCTGGCCACGCAGCTCATGACGCCGGAGAGACGGCGGGCGATCTGGGCGATATACGTGTGGTGCCGGAGGACGGACGAGCTGGTGGACGGCCCGAACGCGGCGCACACGTCGGCGCTGGCGCTGGACCGCTGGGAGTCGCGCCTCGACGGCGTCTTCGCCGGCCGGCCCTACGACATGCTGGACGCGGCGCTGGCCGACGCCGTGGCCGCCTTCCCGGCCGTCGACGAGCGGCCGTTCCGGGACATGGTCCAGGGCATGCGCATGGACCTCGCCAAGTCCCGCTACGCCACCTTCGACGAGCTCTACCTCTACTGCTACCGCGTGGCCGGCACCGTCGGGCTCATGACCGTCCCCGTCATGGGCGTCTCCCCGGGCTCCCAGGCGGACGTCGAGACGGTGTACGCCGGCGCGCTCGCCCTCGGCGTCGCCAACCAGCTCACCAACATCCTCAGGGACGTCGGCGAGGATGCGAGGAGGGGCAGGATCTACCTGCCGCAGAACGAGCTGGCCACGGCCGGCATCTCCGAGGCGGACATCTTCGCCGGCCGCGTCACCGACGAGTGGAGGAGCTTCATGAAGGGCCAGATCGCGAGGGCCAGGGCCTACTTCCAGCAGGCCGAGCAAGGCGCCGCCGAGCTCAACCAGGAGAGCCGATGGCCGGTGTGGGCATCTCTGCTTCTGTACCGTCAGATCCTGGACGAGATCGAGGCCAACGGCTACGACAACTTCAGCAAGCGGGCCTATGTTCCCAAGGCCAAGAAGCTGGCGGCGCTGCCCAAAGCTTACCTCAGATCCCTCATGCCCCCTCCTTCGCAGACGCGCCGTTAA

**>*PSY*3B_Protein**

MLSTGRAVTSPARAAQHRFMGAPRTVVFLAGDHGKRAAASVAARAAGSSHPAVCLEAAAPWSMDCVAAPATVSHEESVRQVVLKQAALAASTTPRKARLAGRDAGVLDAAFARCGAVCKEYAKTFYLATQLMTPERRRAIWAIYVWCRRTDELVDGPNAAHTSALALDRWESRLDGVFAGRPYDMLDAALADAVAAFPAVDERPFRDMVQGMRMDLAKSRYATFDELYLYCYRVAGTVGLMTVPVMGVSPGSQADVETVYAGALALGVANQLTNILRDVGEDARRGRIYLPQNELATAGISEADIFAGRVTDEWRSFMKGQIARARAYFQQAEQGAAELNQESRWPVWASLLLYRQILDEIEANGYDNFSKRAYVPKAKKLAALPKAYLRSLMPPPSQTRR

**Supplemental File S3: *PSY3D gene sequences***

The gene (PSY3D_Gene), CDS (PSY3D_CDS), protein (PSY3D_Protein) sequences are provided as classical FASTA files.

**>*PSY*3D_Gene**

ATGCTCTCCACCGGCCGCGCGGTGACCTCGCCCGCGTGCGCTGCCCAGCGCCGGTTCATGGGCGCGGCGCCAAGGACGGTGGTGTTCCTCGCCGGCGACCACGGGAAGCGAGCTGCTGCTTCTGTGGCAGCGCGCGCCGCCGGCCCGTCCCACCCCGGTGTCTGCCTCGAGGCGGCCGCGCCGTGGAGCATGGAGGACGTCGCGGCGCCGGTGACCGTGTCGTACGAGGAGAGCGTGCGCCAGGTCGTGCTCAAGCAGGCGGCGCTCGCGGCCTCGACCACGCCGAGGAAGGCGCGGCTTTCCAGGCGCGACGCCGGCGTGCTGGACGAGGCCTTCGACCGCTGCGGCGCGGTGTGCAAGGAGTACGCCAAGACGTTCTACCTGGCCACGCAGCTCATGACGCCGGAGCGGAGGCGGGCGATCTGGGCAATATACGGTAATCCACATCGCCACATGACACTGATGCAAGCGACGAGAGGAGATGATTGATGTGGTTACTAACTTCTTGGATGTGTCGACCAGTGTGGTGCCGTAGGACGGACGAGCTGGTGGACGGCCCGAACGCGGCGCACACGTCGGCGCTGGCGCTGGACCGCTGGGAGTCGCGCCTCGACGGCGTCTTCGCCGGCCGGCCCTACGACATGCTCGACGCGGCGCTGGCCGACGCCGTGGCCGCCTTCCCGGCCGTCGACGAGCGGCCGTTCCGGGACATGGTCCAGGGCATGCGCATGGACCTCGCCAAGTCCCGCTACGCCACCTTCGACGAGCTCTACCTCTACTGCTACCGCGTGGCCGGCACGGTCGGGCTCATGACCGTCCCCGTCATGGGCGTCTCCCCGGGCTCCCAGGCGGGCGTCGAGACGGTGTACGCCGGTGCGCTCGCCCTCGGCGTCGCCAACCAGCTCACCAACATCCTCAGGGACGTCGGCGAGGAGTAAGTGTCAGTTCAGTTGTATATATACGTTGCTTCTGAAATCACCTGCTCATCTCTGACGCCGCCGCTGCTGACAGTGCGAGGAGGGGCAGGATCTACCTGCCGCAGGACGAGCTCGCCACGGCCGGCATCTCCGAGGCGGACATCTTCGCCGGTCGCGTCACCGTCGAGTGGAGGAGCTTCATGAAGGGACAGATTGCGAGGGCCAGGGCCTACTTCCAGCAGGCCGAGCAAGGCGCCGCCGAGCTCAACCAGGAGAGCCGATGGCCGGTAAGCGCGCGACAGAAGTTCCATCAGAAAATTCAAAATTCTCAGCCAAAAATGTTAATAGAGTAGTCTAAGTAAGTTTCAGATTAACATGCAATACAAATTGCATGCAGGTGTGGGCATCTCTGCTTCTGTACCGGCAGATCCTGGACGAGATCGAGGCCAACGGCTACGACAACTTCAGCAAGCGAGCCTACGTTCCCAAGGCCAAGAAGCTGGCGGCGCTGCCCAAAGCTTACCTCAGGTCCCTCATGCCTCCTCCTTCTTCGCAGAGGCGCCGTTGA

**>*PSY*3D_CDS**

ATGCTCTCCACCGGCCGCGCGGTGACCTCGCCCGCGTGCGCTGCCCAGCGCCGGTTCATGGGCGCGGCGCCAAGGACGGTGGTGTTCCTCGCCGGCGACCACGGGAAGCGAGCTGCTGCTTCTGTGGCAGCGCGCGCCGCCGGCCCGTCCCACCCCGGTGTCTGCCTCGAGGCGGCCGCGCCGTGGAGCATGGAGGACGTCGCGGCGCCGGTGACCGTGTCGTACGAGGAGAGCGTGCGCCAGGTCGTGCTCAAGCAGGCGGCGCTCGCGGCCTCGACCACGCCGAGGAAGGCGCGGCTTTCCAGGCGCGACGCCGGCGTGCTGGACGAGGCCTTCGACCGCTGCGGCGCGGTGTGCAAGGAGTACGCCAAGACGTTCTACCTGGCCACGCAGCTCATGACGCCGGAGCGGAGGCGGGCGATCTGGGCAATATACGTGTGGTGCCGTAGGACGGACGAGCTGGTGGACGGCCCGAACGCGGCGCACACGTCGGCGCTGGCGCTGGACCGCTGGGAGTCGCGCCTCGACGGCGTCTTCGCCGGCCGGCCCTACGACATGCTCGACGCGGCGCTGGCCGACGCCGTGGCCGCCTTCCCGGCCGTCGACGAGCGGCCGTTCCGGGACATGGTCCAGGGCATGCGCATGGACCTCGCCAAGTCCCGCTACGCCACCTTCGACGAGCTCTACCTCTACTGCTACCGCGTGGCCGGCACGGTCGGGCTCATGACCGTCCCCGTCATGGGCGTCTCCCCGGGCTCCCAGGCGGGCGTCGAGACGGTGTACGCCGGTGCGCTCGCCCTCGGCGTCGCCAACCAGCTCACCAACATCCTCAGGGACGTCGGCGAGGATGCGAGGAGGGGCAGGATCTACCTGCCGCAGGACGAGCTCGCCACGGCCGGCATCTCCGAGGCGGACATCTTCGCCGGTCGCGTCACCGTCGAGTGGAGGAGCTTCATGAAGGGACAGATTGCGAGGGCCAGGGCCTACTTCCAGCAGGCCGAGCAAGGCGCCGCCGAGCTCAACCAGGAGAGCCGATGGCCGGTGTGGGCATCTCTGCTTCTGTACCGGCAGATCCTGGACGAGATCGAGGCCAACGGCTACGACAACTTCAGCAAGCGAGCCTACGTTCCCAAGGCCAAGAAGCTGGCGGCGCTGCCCAAAGCTTACCTCAGGTCCCTCATGCCTCCTCCTTCTTCGCAGAGGCGCCGTTGA

**>*PSY*3D_Protein**

MLSTGRAVTSPACAAQRRFMGAAPRTVVFLAGDHGKRAAASVAARAAGPSHPGVCLEAAAPWSMEDVAAPVTVSYEESVRQVVLKQAALAASTTPRKARLSRRDAGVLDEAFDRCGAVCKEYAKTFYLATQLMTPERRRAIWAIYVWCRRTDELVDGPNAAHTSALALDRWESRLDGVFAGRPYDMLDAALADAVAAFPAVDERPFRDMVQGMRMDLAKSRYATFDELYLYCYRVAGTVGLMTVPVMGVSPGSQAGVETVYAGALALGVANQLTNILRDVGEDARRGRIYLPQDELATAGISEADIFAGRVTVEWRSFMKGQIARARAYFQQAEQGAAELNQESRWPVWASLLLYRQILDEIEANGYDNFSKRAYVPKAKKLAALPKAYLRSLMPPPSSQRRR

**Supplemental Table S1: *PSY3 gene primer sets*.**

As illustrated in the figure 2c, *PSY*3 primer pairs are listed below: in blue, primers designed on *Brachypodium* *PSY*3 sequence and in red, genome-specific primers, used to assign BAC clones to the wheat sub-genomes. Primer pairs in black were used for SSCP and Real-Time PCR analysis.

***Supplemental Table S2: p-value from one-way ANOVA analysis using R software.***

ANOVA p-values are provided for the *PSY*1 to *PSY*3 genes (in column) based on the normalized expression data obtained in leaves and roots (right) at different ABA concentration of 50, 100, 150 μM (left).

***Supplemental Figure S1: PSY3 homoeo-alleles protein sequences.***

Alignment between Renan *PSY*3 protein sequences. Coloured boxes identify motifs predicted by ScanProsite. *PSY*3s share the same motifs identified in *PSY*1 by Crawford [72].

***Supplemental Figure S2: PSY1 and PSY2 expression profile in developing grains.***

*PSY*1 transcript levels at each stage, except at 500DD, are about 2-fold higher than *PSY*2 and between the last two stage there is a significative difference in expression levels while *PSY*2 remains at stable levels during grain development.

***Supplemental Figure S3: PSY3 homoeo-alleles expression during grain development.***

*PSY*3 SSCP profile with primer pair *PSY*3F2R1 is shown, each peak corresponds to one homoeologous copy. In developing grains, transcript levels of A, B and D copies change during the cellular division stages to become then more constant during starch filling. In leaves, the A and the D copies show the higher transcript level, while in roots, D copy represents the major transcript.

***Supplemental Figure S4: PSYs expression after ABA treatment.***

Figure shows expression levels of PSYs in both leaves and roots subjected to 50, 100 and 150µM ABA. Transcript levels were normalized to the signal of wheat RNase L inhibitor-like protein cDNA and expressed relative to the level detected in not stressed leaves and roots.


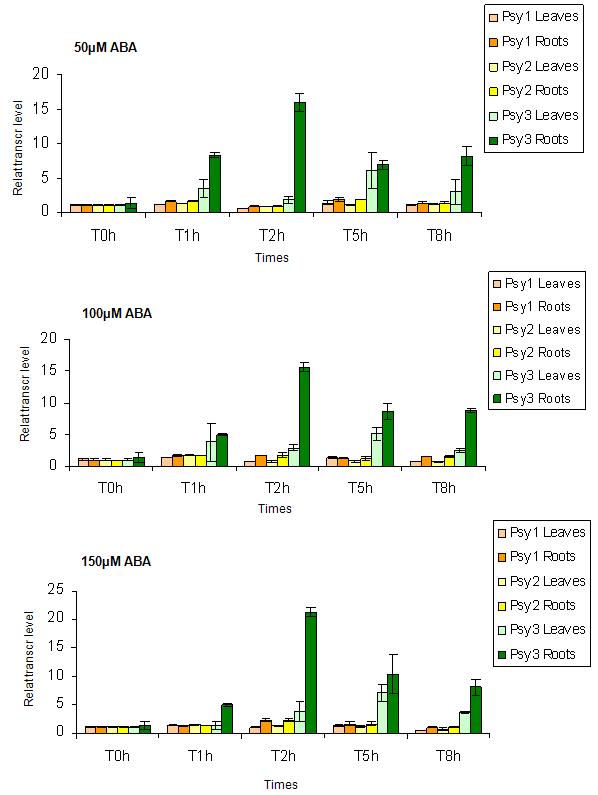

Supplement: Additional file 1 — S1.PSY3A gene sequences. S2.PSY3B gene sequences. S3.PSY3D gene sequences. Table S1.PSY3 gene primer sets. Table S2. p-value from one-way ANOVA analysis using R software. Figure S1.PSY3 homoeo-alleles protein sequences. Figure S2.PSY1 and PSY2 expression profile in developing grains. Figure S3.PSY3 homoeo-alleles expression during grain development. Figure S4. PSYs expression after ABA treatment. [file 1471-2164-13-221-S1.doc]
